# Risk-Stratified Pathways for Cancer Survivorship Care: Insights from a Deliberative Multi-Stakeholder Consultation

**DOI:** 10.3390/curroncol28050295

**Published:** 2021-09-05

**Authors:** Dominique Tremblay, Nassera Touati, Karine Bilodeau, Catherine Prady, Susan Usher, Yves Leblanc

**Affiliations:** 1 Faculté de Médecine et des Sciences de la Santé, Campus de Longueuil-Université de Sherbrooke, 150 Place Charles-Le Moyne, Longueuil, QC J4K 0A8, Canada; catherine.prady@usherbrooke.ca (C.P.); susan.elizabeth.usher@usherbrooke.ca (S.U.); 2 Centre de Recherche Hôpital Charles-Le Moyne, Centre Intégré de Santé et de Services Sociaux de la Montérégie-Centre, Longueuil, QC J4K 0A8, Canada; yves.leblanc3@usherbrooke.ca; 3 École Nationale d’Administration Publique, 4750 Henri-Julien Avenue, Montréal, QC H2T 3E5, Canada; nassera.touati@enap.ca; 4 Faculté des Sciences Infirmières et Centre D’Innovation en Formation Infirmière, Université de Montréal, Montréal, QC H3T 1A8, Canada; karine.bilodeau.2@umontreal.ca; 5 Centre Intégré de Cancérologie de la Montérégie, 3120 Boulevard Taschereau, Greenfield Park, QC J4V 2H1, Canada

**Keywords:** risk stratification, survivorship care, deliberative consultation, multi-stakeholder, care coordination

## Abstract

Risk-stratified pathways of survivorship care seek to optimize coordination between cancer specialists and primary care physicians based on the whole person needs of the individual. While the principle is supported by leading cancer institutions, translating knowledge to practice confronts a lack of clarity about the meaning of risk stratification, uncertainties around the expectations the model holds for different actors, and health system structures that impede communication and coordination across the care continuum. These barriers must be better understood and addressed to pave the way for future implementation. Recognizing that an innovation is more likely to be adopted when user experience is incorporated into the planning process, a deliberative consultation was held as a preliminary step to developing a pilot project of risk-stratified pathways for patients transitioning from specialized oncology teams to primary care providers. This article presents findings from the deliberative consultation that sought to understand the perspectives of cancer specialists, primary care physicians, oncology nurses, allied professionals, cancer survivors and researchers regarding the following questions: what does a risk stratified model of cancer survivorship care mean to care providers and users? What are the prerequisites for translating risk stratification into practice? What challenges are involved in establishing these prerequisites? The multi-stakeholder consultation provides empirical data to guide actions that support the development of risk-stratified pathways to coordinate survivorship care.

## 1. Introduction

A key challenge in cancer care delivery is the complexity of involving multiple care providers over long time periods now that many cancers are experienced as a chronic disease [1,2,3,4]. Cancer care models recognize the importance of self-management support, vigilant attention to signs of health status deterioration and timely access to primary or specialized care when needed [5,6]. Some people living with and beyond cancer (cancer survivor in this article) have complex health conditions that require high intensity ongoing care from specialized cancer teams, while others are able to meet their needs with minimal specialist involvement [7]. Risk-stratification is a way to align services with the evolving health status of cancer survivors and help them navigate across a coordinated continuum without getting lost during transitions [8].

Risk-stratified models are a means of designing evidence-based comprehensive coordinated care that is tailored to the specific needs of the individual and uses healthcare resources appropriately. Coordinated models involve sharing information and responsibility and clarifying tasks [9], which, over the continuum, present challenges related to the cost, complexity and chronicity of cancer care [10]. Risk-stratified survivorship care models are promoted to benefit survivors and clinicians, especially regarding coordination between specialized and primary care [11]. This model of care has been on the agenda for some 15 years, but it has yet to be implemented in many healthcare systems [12,13,14,15]. Early experiments suggest that this model of follow-up care has potential to better adapt care to individual needs and improve decision-making and health services utilization [16]. This article presents findings from a deliberative consultation that sought to understand the perspectives of multiple actors—including cancer survivors—on barriers to risk-stratified survivorship care in order to reduce the knowledge to practice gap.

While there has been some experimentation with risk-stratified models, they have yet to be widely adopted in practice. A model of longitudinal risk-based care was proposed in the seminal work of Oeffinger that focused on survivors of childhood cancers and was adapted in adult populations to prevent survivors from getting lost in transition [17]. The model assumed that improvements in outcome and quality of care would come from screening and surveillance of the late effects of cancer treatments. This work paved the way for long-term survivorship care shared between cancer specialists and primary care physicians. Oeffinger’s model identified multiple potential barriers and enablers related to the characteristics of health systems (e.g., healthcare policies, insurance coverage), healthcare providers (e.g., knowledge of late effects, organization of practice, attitudes towards cancer survivorship) and survivors (e.g., cancer experience, knowledge of risks, socio-demographic status, cues to action) that remain relevant for survivors overall. Recent work has advocated risk-stratified follow-up for cancer survivors as a way to meet patients’ individualized needs, increase patient satisfaction with care and decrease healthcare-related costs [18]. In stratifying pathways, risk is assessed as low, moderate or high according to the type of cancer treatment, potential late treatment-related effects as well as the risk of recurrence [14]. A comprehensive assessment of patient needs and risk inform individualized risk-stratified follow-up plans [8,14,19]. Some authors recommend that cancer patients at higher risk be mainly followed by the cancer care team, while patients at moderate risk be mainly followed by primary care providers [20] and those at low risk receive self-management support accompanied by facilitated access to cancer services re-entry [18,20].

For example, in the UK, risk-stratified cancer survivorship pathways have been implemented for patients with breast, prostate and colorectal cancers [21]. Risk stratification starts immediately after diagnosis with a holistic assessment of physical, psychological and social needs. Individualized self-management support might be sufficient for cancer survivors at low risk of recurrence, treatment toxicity or late treatment-related effects, especially when they also have few other health needs. A coordinated care approach would be preferred for cancer survivors who face medium risk of recurrence and late effects and have other health needs, while follow-up primarily by a specialized cancer team might be appropriate for patients with high probability of complex needs, recurrence or late cancer treatment effects. In addition, cancer survivors are assigned formal contact points to ensure timely access to cancer care if needed [22]. A risk-stratified pathway may include a combination of self-management support, fast re-access to the system, scheduled screening for recurrence, follow-up with a clinical nurse specialist, along with clinical support services such as psychological, continence, diet and nutrition counselling. Other general supports such as social services and vocational rehabilitation can also be integrated into a personalized risk-stratified pathway [23]. The risk-stratified approach may promote patient awareness about warning symptoms of late effects and recurrence, and experience to date in the UK suggests that it improves patient experience and patient satisfaction with potentially lower follow-up costs [23].

Meeting both the medical and psychosocial needs of cancer survivors and implementing risk-stratified pathways, relies on individualized care coordinated between specialized cancer teams, primary care providers and self-management support. However, many practitioners lack training in cancer risk stratification [24], support for self-management remains under-developed and coordination is insufficient to ensure that survivors do not get lost in transition. Better understanding of these barriers is essential for the development of care pathways based on risk. 

This paper reports on a deliberative consultation designed to incorporate provider and user perspectives and experience into the planning of a larger study to develop, test and measure outcomes of a risk-based coordinated cancer care model for patients transitioning from specialized oncology teams to primary care providers. The deliberative consultation is selected based on the research team’s belief that bringing together knowledgeable actors and researchers can produce insights into effective practice transformation in cancer care. Innovations are more likely to be adopted when user experience is incorporated into the planning process [25]. In deliberative consultation, participants are provided with information (definitions, mechanisms, outcomes) about the risk-stratified model and are invited to discuss and challenge various points of view and recommend potential targets for implementation. Researchers can identify patterns in these deliberations.

Deliberative consultation builds on the Nose to Tail (NTT) approach [26], designed to support the development, implementation and scale-up of innovations in health care (a risk-stratified survivorship care model in our case). NTT helps think through the feasibility of an innovation at an early stage of planning and stresses that introducing new ways of doing things in the healthcare system is more likely to be successful when the experience of final users is incorporated into the planning process. NTT exercises proceed step by step through a series of stages and questions. The deliberative consultation covers the first three stages suggested in the NTT model: identify a shared definition of risk stratification, determine the prerequisites for translating risk stratification into practice and overcoming barriers, and identify resources needed to test a risk-stratified model of follow-up care for cancer survivors. 

Building on initiatives underway in other countries [27], the deliberative consultation addresses three main questions: (1) What does a risk-stratified model of cancer survivorship care mean to care providers and users? (2) What are the prerequisites for translating risk stratification into practice? (3) What challenges are involved in establishing these conditions? Findings are intended to assist clinicians and policymakers achieve delivery models that help address important challenges in contemporary survivorship care.

## 2. Materials and Methods

### 2.1. The Deliberative Method

The deliberative consultation is part of a larger realist evaluation of practice in cancer survivor transitions between specialized oncology and primary care teams [9].

### 2.2. Procedure

The deliberative consultation was held on two sites located in two regions in Quebec (Canada), which has a publicly funded healthcare system. The sites were selected for their geographic diversity. The first (Site A) is an urban territory of 1391 km^2^ providing cancer care to 420,000 people. The second (Site B) is a remote territory of 95,762 km^2^ providing cancer care to a population of 277,796. Team members with clinical expertise in cancer care (K.B., D.T. and C.P.) and members with expertise in organizational models of cancer care (D.T. and N.T.) were present at each consultation. The academic researchers had expertise in facilitating research group discussions in cancer care (K.B., N.T.) and primary care (N.T.) and in leading (D.T.) a research program on complex interventions in oncology that involved more than 20 group discussions with decision-makers, professionals and cancer patients. C.P. is a cancer specialist, researcher and administrator in a cancer center. K.B. and N.T. facilitated discussion at Site A, and D.T. and C.P. facilitated the consultation at Site B. These dyads encouraged the active participation of all stakeholders. Systematic observation of participants non-verbal reactions and reflection about answers to N.T.T. questions was undertaken by note-takers—two research assistants with experience in discussion groups—from the research team at each site. The half-day consultation, held simultaneously on both sites, began with a presentation by D.T. and C.P. on risk-stratified pathways (transmitted by videoconference to Site A) that described the objectives of the consultation and provided a synthesis of the literature on risk-stratified models in cancer care delivery. Table 1 summarizes the material presented to participants. Small group deliberation at each site was followed by mixed-group deliberation around key points raised at each site. 

### 2.3. Participants

A snowball sampling technique [29] was used to identify stakeholders from cancer teams and primary care who were knowledgeable about the cancer care trajectory, the clinical and social environment, existing healthcare coordination mechanisms and anticipated barriers and opportunities (Table 2). Cancer survivors with experience on various governance and improvement committees were also identified to assure inclusion of user perspectives [30]. A total of 37 stakeholders were invited and 25 agreed to participate in the consultation; one oncologist cancelled at the last minute, leaving a total of 24 stakeholders along with 6 research team members; of the total 30 participants, 80% were women. The study was approved by the Research Ethics Committee of Centre intégré de santé et de services sociaux de la Montérégie-Centre-Hôpital-Charles Le Moyne (CE-HCLM-17-027) and was conducted in accordance with both national ethical policy for research involving humans [31] and the local standards of participating sites.

### 2.4. Data Collection

The qualitative data collection grid was based on the following questions drawn from the NTT tool [27]: What are your thoughts on the definition of risk stratification? How might this concept be adopted in the current practice environment? Which actors need to be involved in the adoption and implementation process? What do you see as the main barriers and facilitators to implementing a risk-stratified model? Are current conditions in the system favorable to implementing this type of innovation? What are the continuing education and coordination mechanisms required to ensure an effective risk-stratified approach? Discussions were audio recorded and transcribed for further analysis. Note-takers on each site collected handwritten observation notes [32] These raw data were all input into a formal database and managed with QDA Miner^TM^ (version 5.0.34) [33] and stored securely.

### 2.5. Analysis

Descriptive interpretation [34] focused on data condensation using a 3-cycle coding strategy to analyze participant contributions to the deliberations [32]: the first descriptive coding assigned labels to raw data to reduce content to a word or a short phrase, a second cycle organized content into categories, and a third aggregated content into themes related to the consultation questions. Two team members (D.T. and Y.L.) independently performed the first coding cycle and the two other cycles involved discussion among the research team members to reach consensus. Rigor and transparency were assured with attention to confirmability, reliability, credibility and transferability [32]: (1) DT kept a research diary to document the data collection experience and keep methodological and interpretative notes; (2) interview transcripts were validated against the digital audiotapes; (3) the coding cycles of the two focus groups and joint plenary session were performed independently by YL and DT, then discussed with a research professional (SU) to reach consensus. Finally, the aggregated themes were validated with the co-authors; and (4) participant characteristics were described. 

## 3. Results

The deliberations documented stakeholders’ perceptions of the risk-stratified pathways, the prerequisites for putting risk stratification into practice and the conditions and resources that might be required to lay the groundwork for change. While there were some minor differences between the two regions, the findings presented here represent perspectives voiced in both sites.

Our central finding is that translating risk-stratified pathways for cancer survivors belongs to the category of wicked problems, which are difficult to understand, ill-formulated, linked to multiple other underlying problems and have complexities that are underestimated for which solutions are not readily apparent [35]. Risk stratification for cancer survivors appears difficult to define, involves multiple actors with frequently competing mental models, and there is no immediate solution for practice. Like any wicked problem, the more stakeholders explored solutions, the more underlying problems appeared [36]. Three main themes emerge from the analysis: conceptual ambiguity, overcoming ill-defined and organizational monoliths, laying the groundwork. The next sections present these themes in more detail.

### 3.1. Conceptual Ambiguity

Our first question was: What does a risk stratified model of cancer survivorship care mean to care providers and users? This question proved difficult because most participants had not heard the term before the deliberative consultation. Discussion focused on the notion of risk, distinguishing risk from cancer survivor needs and responsibility for minimizing risk, pointing to areas that require clarification before risk stratification can be understood and used. All participants agreed on the need to improve transitions along the cancer trajectory. “Right up until I saw my family doctor a year and a half later (…) there was a lack of attention to the risk of side effects” (cancer survivor, Site B). However, some insisted on the need to distinguish between risk stratification and evaluation of survivor needs and/or unmet needs. The process of risk assessment and stratification to guide pathways was seen to require more clearly establishing how—and by whom—responsibility is assumed for follow-up care and support for self-management and health system navigation. “There are definitely gaps that need to be addressed first [to enable risk stratification]” (Oncology nurse, Site B). Participants stressed that the notion of risk is “evolutive”, considering the variable evolution of the disease at individual level and fast-advancing science, raising questions around how often risk assessment would be needed. “The way I saw a patient with lung cancer 5 years ago is completely different from how I see them today; this concept (of oncologic risk) has to evolve along with the science” (Family physician, Site A). 

A main challenge identified by participants was the “lack of a clear definition of oncologic risk in nursing and psychosocial practice” (Social worker, Site A). Very few providers undertake risk assessments, mainly because they are unfamiliar with the concept in the context of establishing pathways. “I still have trouble understanding the concept; the definition isn’t clear (…) maybe the definition isn’t quite right and should be reworked, repositioned” (Primary care manager, Site B). 

### 3.2. Overcoming Ill-Defined Roles and Organizational Monoliths

Our second question was: what are the prerequisites for translating risk stratification into practice? Discussion around existing care provider roles drew a portrait of oncology care as a “monolith”—a massive column standing alone (Observation note-taker, Site A). Participants mentioned limitations to the type of coordination between providers needed to enact individualized follow-up based on risk. Limitations had to do with role definition, information transmission and disruption of primary care during cancer treatment. The role of care coordinator is assumed by the pivot nurse (also called nurse navigator or nurse coordinator) in oncology embedded within the cancer care team, but that role is tightly circumscribed from extending farther across the trajectory. Administrative rules related to case load make it difficult to assure smooth transitions along the entire cancer trajectory; for example: “the cancer care team is responsible for providing psychosocial services over a fixed duration (6 months) following the end of active treatment” (Oncology Nurse, Site B). 

Participants considered that operational challenges in information transmission and role definition complicate adoption of risk-stratified pathways: care transitions between specialists and primary care are not optimal because information systems are not connected and survivorship care plans are not in common use. Primary care physicians might have access to information about the patient’s treatment course, but have no direct contact with the cancer team and no communication about what role they are expected to play in addressing survivorship issues that might emerge. As one participant said: “As a family physician, I receive a summary once my patient sees an oncologist, which indicates that the patient has been seen, but no information that clarifies my own role in following the patient” (Family physician, Site B). Another stated: “When the patient is in the oncology ward, we don’t see him any more until the end of cancer treatment. The family physician receives no information regarding the treatments. There is no continuity of care. This type of hand-off is a real problem” (Family physician, Site A).

Primary care is seen to play a key role in the accurate and timely assessment of symptoms for early diagnosis, but has an ill-defined role from the investigation stage onward. Participants saw the main reason for this as a lack of knowledge regarding practices tailored to cancer survivors, which works against the development of expertise and reduces physician, nurse, and social worker confidence in their ability to intervene. “The Family Medicine Group (FMG) nurses, if you asked them to undertake follow-up for a cancer survivor, they would say: I don’t know what to do” (Primary care nurse, Site A).

Knowledge of follow-up survivorship plans appears as a necessary but not sufficient condition for change. Both during and after active treatment, closer collaboration between teams is required for primary care providers to assure care that is suitable for the level of risk, while being able to transition the patient back to specialist care when needed. The lack of effective coordination between cancer teams and primary care providers was mentioned as a main impediment to developing risk-stratified pathways for cancer survivors. Patient participants in the deliberative consultation agreed with this assessment, finding that there is little communication or cohesion among specialists within the cancer team, or between these specialists and family physicians. Very often, no pertinent up-to-date information is transmitted regarding decisions about a patient’s cancer care and follow-up, or about their general state of health. “They (clinicians) have to talk to each other: at one point, the oncologist prescribed a medication for me, which the urologist then cancelled. Why did he do that? We don’t know what to say or what to do” (cancer survivor, Site B). Cancer survivor participants also mentioned uncertainty about what relationship they should maintain with primary care providers during and after cancer treatment. Participants reported that few survivors consult their primary care provider after they receive a cancer diagnosis: they already have too many appointments and are reluctant to impose an additional workload on providers.

Participants considered that the lack of communication resulted from the “monolithic functioning” within and between care teams. Providers do not know the other providers called upon to intervene in a cancer survivor’s trajectory, much less the roles they play in that care. The lack of role clarity impedes information exchange and effective coordination of care and services between teams.

### 3.3. Laying the Groundwork

The third question discussed in the consultation was: what challenges are involved in establishing these prerequisites? The discussion provided a valuable starting point to delve deeper into issues that impede the redefinition and redistribution of roles foreseen in a risk-stratified pathway. The three main issues raised in the consultation were primary care capacity to confidently assume greater responsibilities in risk-stratified pathways, timely and appropriate communication between primary care and specialized teams and cancer survivor role in relaying information to their primary care provider. The portrait also pointed to steps that could be taken to lay the groundwork for innovation and practice transformation.

To increase primary care capacity, participants suggested “offering continuing education for primary care providers to keep up their knowledge of oncology practice and provide specific training in oncologic risk approaches” (Oncologist, Site A). They pointed to existing continuing education structures that could integrate this training. Enhancing knowledge might enable better coordination of care and increase the participation of various actors in a risk-stratified model.

To improve coordination between specialist and primary care teams, participants proposed a number of possible short-term solutions. A first would be “to open up participation in (oncology) sector committees to other providers who are expected to play a role in the care of cancer survivors” (Family Physician, Site A). This would enable providers to understand the role each plays in follow-up care. Another would be to create ad hoc working groups to bring these actors together to discuss particular complex cases encountered in cancer survivor follow-up. The regular regional FMG leaders’ meetings and annual conferences were suggested as promising venues to bring cancer teams and primary care providers together. Opportunities for exchange were seen as “essential to creating trust relationships between providers” (Oncology nurse, Site A).

A third suggestion from participants was to enhance the capacities of cancer survivors. In the current context, a large share of responsibility for navigating cancer care and follow-up, transmitting information between cancer team and primary care and, in effect, determining how involved primary care will be in their cancer care and follow up is left to the cancer survivor. Participants recognized (1) that this places an undue burden on survivors; (2) that cancer survivors could be much better equipped to play this role and (3) that cancer teams often actively discourage survivors from involving their primary care provider. Cancer survivor participants felt they had a major role in relaying information, but lacked clear and consistent guidance on what they should be reporting. There would appear to be an opportunity for patient education to reinforce expectations of shared care. Survivors need support to play a meaningful role in risk assessment. “Most of the information is transmitted by the patient. We may have to think about how to equip patients to transmit information more effectively.” (Cancer survivor, Site A).

The final segment of the consultation elicited suggestions from participants about measures that would help lay the groundwork for translating knowledge to practice. Enhancing the capacities of primary care providers is crucial but not sufficient: the organization of services, access corridors and self-management supports are equally important to understanding and implementing risk-stratified pathways. 

## 4. Discussion

To the best of our knowledge, this deliberative consultation was the first to be held in Québec, which has a publicly funded healthcare system and has been developing its cancer care network for over 20 years. The consultation revealed that the implementation of risk-stratified pathways for follow-up care of cancer survivors is a wicked problem, that the concept is not well known, and that efforts will be needed to counter rigidities in the system, more clearly define roles, create links between care settings (joint meetings, committees, training) and more actively involve cancer survivors at an early stage.

Positive experience in the UK with risk-stratified pathways has encouraged efforts by other countries, notably to improve sustainability [11]. However, the concept raises some conceptual and practical concerns. It hinges on the ability to develop a common understanding of “risk”, which is difficult for two main reasons. First, few models are available for the early detection or management of late effects that would be needed for risk-stratified survivorship care [37] Second, even if such models were available, many factors other than risk of late treatment effects or recurrence are involved in determining survivorship care needs and the types of providers involved in that care. Alfano et al. (2019) find that the language of “risk-stratified care” is evolving towards one of “personalized pathways” [25] (p. 235). From a different angle, Pizzoli et al. (2019) raise concerns about the concept of “chronic cancer” [38] that can signal use of alternate follow-up care approaches and emphasis on self-management. They underline that unlike chronic conditions such as diabetes or asthma, there is little certainty about when and if cancer becomes “chronic”. It denotes a vast space between cured, risk-stratification and survivorship that can produce confusion for both cancer survivors and care providers. Participants in the deliberative consultation had little pre-existing knowledge of risk-stratified pathways, but the ambivalence they expressed in the discussion reflects the above concerns. 

In terms of how to operationalize approaches to follow-up care that consider primary care and cancer survivor self-management capacities as potential solutions, the challenges appear much the same whether “risk” or “personalized” terminology is used. They involve change from multiple different actors: oncology specialists, general practitioners, other professionals, community organizations, patients and families. Multiteam systems theory, taken from organization science [39], considers that this translation is undertaken “by integrating the work of specialized ‘component’ teams” to offer comprehensive solutions to complex problems (Ascencio 2012, p. 487). It involves working towards both goals specific to a given “team” and the wider goal they are all working towards. Literature on multiteam systems in the health field points to the importance of establishing communication norms, distributed leadership mechanisms, and using boundary-spanning actors to move between teams and help to “coordinate collective action” (Ascencio 2012, p. 493). However, many questions remain about how to support multiteam improvement efforts [40].

The preoccupation, in the deliberative consultation, with communication between specialized cancer teams and primary care providers reflects findings from a consultation held by the Canadian Team to Improve Community-Based Cancer Care Along the Continuum in 2017, where participants looking at ways to improve coordination prioritized implementation of an eConsult platform for communication between family physicians and cancer specialists [13]. Improving the capacities and confidence of primary care providers was also highlighted in the present deliberative consultation. Chaput and Sussman [10] point to survivorship training resources for primary care providers developed in several Canadian provinces (BC, Ontario, Manitoba), as well as opportunities for inter-specialty education, as promising steps. This may contribute to establishing a cancer survivor’s level of risk by raising awareness of the range and potential severity of effects of cancer and its treatment. 

As partners with healthcare teams, cancer survivors play a significant role in coordinating follow-up care [41], notably in the context of self-management and communication between healthcare team members. Participants in the deliberative consultation considered that patients should be better equipped for, and recognized in, this role. Integrating self-management supports was an important feature of UK risk-stratified follow-up models [21]. The Global Partners on Self-Management in Cancer, a sub-group of the Multinational Association Supportive Care in Cancer, recently put out a call for action in six priority areas: prepare patients to be actively involved, shift the care culture to embed self-management in care pathways, train the workforce to support self-management, establish patient-reported outcome measuring, advance evidence on self-management and self-management support in cancer and expand access to self-management support programs [5]. Authors recognize that building capacity requires action on multiple levels, from patients and their support networks, to provider practices and teams, to healthcare organizations, all the way to policies and financing [5,10]. The deliberative consultation described in this article reveals that the participation of cancer survivors adds important dimensions. 

Finally, the deliberative consultation did not provide information about the type of leadership that would be needed to implement this approach. Prior research undertaken in the Quebec Cancer Network provides some insight in this regard. “Leadership that is shared to ensure the quality of care during transition also entails a boundary-spanning process that transcends the traditional division of professional labor in a health care system with function-based organizational boundaries” [42] (p. 1014). While enacting shared leadership is a complex and ongoing process, it appears crucial for supporting the implementation of risk-stratified pathways.

## 5. Strengths and Limitations

The participation of knowledgeable informants provided agreement on potential barriers to be addressed before implementation of a new model of risk-stratified pathways for cancer survivors. Participants in the deliberative consultation were selected for their extensive lived experience in cancer and survivorship care and varied perspectives, however other providers and cancer survivors may have divergent views and additional insights. It is difficult to ensure that discussion among a small group reflects larger perspectives in the field [25]. Additionally, given the considerable barriers to implementing a risk-stratified model despite evidence of its benefits, an important question the present study does not consider is how decision-makers weigh the recommendations from research against political and resource considerations. Our decision not to include top managers and policy-makers was taken out of concern for power differentials that might hinder open discussion.

## 6. Conclusions

The deliberative consultation reveals that risk-stratified pathways serve as a valuable trigger for discussion about models of survivorship care; however, translating the concept into practice and assuring its contribution to value-based care remains a challenge.

Findings from the deliberative consultation provide valuable information on the groundwork that needs to be laid before attempting to implement a risk-stratified model of follow-up care for cancer survivors. Participant contributions help to understand the environment and local context in which the model is expected to take shape, as well as identifying stakeholders who need to be involved in its implementation. These results have value outside the particular context of regional networks in Quebec and point to the considerable work that remains to be done to adapt cancer care to contemporary challenges, making best use of available resources to produce outcomes of value. 

## Figures and Tables

**Table 1:** Material presented to participants in the deliberative consultation.

Definition of risk stratification: “the process of quantifying the probability of a harmful effect to individuals resulting from a range of internal and external factors (e.g., demographic characteristics, genetic make-up, medical treatments). (…) Risk must be differentiated from (healthcare) need, which is the capacity to benefit from health care. A need must be present at the time of assessment, unlike a risk, which implies something that might happen in the future” [8]
Timing of follow-up visits: Risk-stratified follow-up involves assessing patients following initial treatment and assigning a level of risk that informs an individual follow-up care plan [28].
Elements to be documented in assessing risk include: type of cancer, type of treatment, potential late treatment-related effects, signs of recurrence or new cancer, signs and symptoms of chronic diseases and state of emotional health [14]. A survivorship care plan should be in place but is often not [15].
The literature suggests that assessment likely requires a core tool relevant to most cancers, with specific additions for different tumor types.
The framework for risk stratification [8] recognizes that risks will vary over time, requiring repeat assessment: the first time point would be following diagnosis and pre-treatment, and the second on completion of primary treatment. Further assessments may be required as needs change over time [28].

**Table 2:** Participants.

Participants	Site A	Site B	Total (*n* = 30)
Managers	3	3	6
Allied health professionals	3	2	5
Family physicians	1	3	4
Professors (Faculty of Medicine)	2	1	3
Oncologists	1	1	2
Cancer survivors	2	1	3
Ph.D. Student	1	0	1
Total stakeholders	13	11	24
Research team members	3	3	6
Total participants			30

## Data Availability

Not applicable.
